# Impaired Ca^2+^ Signaling in β-Cells Lacking Leptin Receptors by Cre-loxP Recombination

**DOI:** 10.1371/journal.pone.0071075

**Published:** 2013-08-01

**Authors:** Eva Tudurí, Jennifer E. Bruin, Heather C. Denroche, Jessica K. Fox, James D. Johnson, Timothy J. Kieffer

**Affiliations:** 1 Department of Cellular and Physiological Sciences, Life Sciences Institute, University of British Columbia, Vancouver, British Columbia, Canada; 2 Department of Surgery, University of British Columbia, Vancouver, British Columbia, Canada; Universidad Miguel Hernández de Elche, Spain

## Abstract

Obesity is a major risk factor for diabetes and is typically associated with hyperleptinemia and a state of leptin resistance. The impact of chronically elevated leptin levels on the function of insulin-secreting β-cells has not been elucidated. We previously generated mice lacking leptin signaling in β-cells by using the Cre-loxP strategy and showed that these animals develop increased body weight and adiposity, hyperinsulinemia, impaired glucose-stimulated insulin secretion and insulin resistance. Here, we performed several *in vitro* studies and observed that β-cells lacking leptin signaling in this model are capable of properly metabolizing glucose, but show impaired intracellular Ca^2+^ oscillations and lack of synchrony within the islets in response to glucose, display reduced response to tolbutamide and exhibit morphological abnormalities including increased autophagy. Defects in intracellular Ca^2+^ signaling were observed even in neonatal islets, ruling out the possible contribution of obesity to the β-cell irregularities observed in adults. In parallel, we also detected a disrupted intracellular Ca^2+^ pattern in response to glucose and tolbutamide in control islets from adult transgenic mice expressing Cre recombinase under the rat insulin promoter, despite these animals being glucose tolerant and secreting normal levels of insulin in response to glucose. This unexpected observation impeded us from discerning the consequences of impaired leptin signaling as opposed to long-term Cre expression in the function of insulin-secreting cells. These findings highlight the need to generate improved Cre-driver mouse models or new tools to induce Cre recombination in β-cells.

## Introduction

A major contribution to the rise of diabetes is the increasing incidence of obesity; approximately 80% of the diabetic patients are obese [1]. While the molecular mechanisms for the obesity-diabetes link remain unclear, the adipocyte hormone leptin seems to be a key factor. Leptin is well known for decreasing food intake and increasing energy expenditure via signaling through its receptor in the hypothalamus [2]. In addition, leptin receptors are also expressed in peripheral tissues, as well as the endocrine pancreas [3], [4] where leptin inhibits the expression and secretion of insulin [5], [6], [7] and glucagon [4], [8]. Obese rodents and humans generally display increased circulating leptin levels [9], [10]; however, despite the hyperleptinaemia, leptin often fails to cease obesity development [9], [10], [11]. Such a failure of leptin to suppress appetite and body weight gain is considered evidence for leptin resistance in the hypothalamus [12], [13]. Moreover, leptin resistance in peripheral tissues has been observed in diet-induced obese mice [14]. It has also been proposed that leptin resistance in β-cells could be involved in the abnormal response of these cells to sustained hyperglycemia [15], [16]. Leptin deficient (*ob/ob*) and leptin receptor deficient (*db/db*) mice exhibit obesity, hyperglycemia, hyperinsulinema and hyperglucagonemia [17], [18], [19]. Moreover, disruption of leptin receptors in β-cells results in increased body weight and adiposity, hyperinsulinemia, impaired glucose-stimulated insulin secretion and insulin resistance [16]. Hence, these observations suggest that leptin resistance in β-cells may contribute to obesity-related diabetes.

In this study we aimed to unravel the underlying mechanisms by which pancreatic β-cells deficient in leptin signaling over-secrete insulin under basal conditions and fail to properly secrete insulin in response to glucose. For that purpose, we employed mice that lack leptin receptors in β-cells, referred to as *Lepr^flox/flox^ RIP-Cre* mice, by means of the Cre-loxP recombination approach. In the context of diabetes, mice expressing Cre recombinase under the rat insulin II promoter (commonly known as *RIP-Cre* mice) are widely used for specifically knocking down genes within the β-cell population. However, *RIP-Cre* mice have been reported to develop glucose intolerance and impaired insulin secretion [20]. In contrast, we [16] and others [21] have not observed any impairment in glucose clearance and insulin release when using *RIP-Cre* mice, which suggests that the abnormalities in the glucose excursions are strain related, as previously reported [20], [21].

Here, the examination of β-cells from *Lepr^flox/flox^ RIP-Cre* mice revealed several abnormalities in the stimulus-secretion coupling and intercellular communication, as well as an increase in autophagic degradation of insulin secretory granules. Notably, defects in intercellular communication were observed within neonatal *Lepr^flox/flox^ RIP-Cre* β-cells prior to any metabolic phenotype. Unfortunately, these studies also revealed that despite exhibiting normal glucose tolerance and GSIS, control mice homozygous for the wild type leptin receptor allele and carrying the Cre transgene (*Lepr^+/+^ RIP-Cre* mice) displayed abnormal β-cell intracellular Ca^2+^ oscillations making it difficult to discern the contribution of impaired leptin signaling to the *Lepr^flox/flox^ RIP-Cre* β-cell phenotype. Therefore, we urge caution in the use of *RIP-Cre* mice for inducing Cre recombination in insulin-secreting β-cells.

## Materials and Methods

### Ethics Statement

All procedures with animals were approved by the University of British Columbia Animal Care Committee (protocol A10-0275) and carried out in accordance with the Canadian Council on Animal Care guidelines.

### Animals

*Lepr^flox/flox^ RIP-Cre* mice were generated by crossing C57BL/6-TgN(Ins2-Cre)25 Mgn [22] with *Lepr^flox/flox^* mice [23], as previously described [16]. *Lepr^+/+^ RIP-Cre* mice were generated from the heterozygous offspring produced by intercrossing *Lepr^flox/flox^ RIP-Cre* mice with C57BL/6 mice (obtained from the Jackson Laboratory, Bar Harbor, ME). All mice employed in this study were male, and were housed with a 12 h light, 12 h dark cycle and had ad libitum access to chow diet (5015, Lab Diet, St Louis, MO) and water.

### Islet Isolation

Hank’s balanced salt solution (HBSS) containing 1000 U/mL of type XI collagenase (Sigma-Aldrich, St. Louis, MO) was used to isolate both neonatal and adult islets. Neonatal pancreases were harvested, quickly minced in the collagenase-containing solution, and digested at 37°C for 5 min. Adult pancreas were injected with the collagenase solution by the duct prior to their removal and digested at 37°C for 11 min. Islets were then washed with iced-cold HBSS and handpicked under a microscope.

### Imaging

Intact pancreatic islets were imaged following 36–48 h culture at 37°C and 5% CO_2_ on glass coverslips. Coverslips were transferred to an imaging chamber mounted on a temperature-controlled stage and held at 37°C on a Zeiss Axiovert 200 M inverted microscope equipped with a FLUAR 10X objective (Carl Zeiss, Thornwood, NY). During the experiments, islets were continuously perifused with Ringer's solution containing (in mM): NaCl 144, KCl 5.5, CaCl_2_ 2, MgCl_2_ 1, Hepes 20 (adjusted to pH 7.35 by NaOH). For intracellular Ca^2+^ measurements, islets were loaded with 5 µM Fura 2-AM for 30 min. The wavelengths of fluorescent excitation were controlled by means of excitation filters (Chroma Technology, Rockingham, VT) mounted in a Lambda DG-4 wavelength switcher (Sutter Instrument Company, Novato, CA). Fura-2 AM was excited at 340 nm and 380 nm and the emitted fluorescence was monitored through a D510/80 m filter. Changes in [Ca^2+^]_i_ were expressed as the ratio of the fluorescence emission intensities (F340/F380). NAD(P)H levels were monitored as changes in autofluorescence using a D365/10x excitation filter and a 445 nm emission filter. Images were acquired using a CoolSNAP HQ digital camera (Roper Scientific, Tucson, AZ). Slidebook software package (Intelligent Imaging Innovations, Inc. Denver, CO) was employed for image acquisition and imaging analysis.

### Transmission Electron Microscopy

Harvested pancreata were immediately cut into small pieces and fixed in 2% glutaraldehide at 4°C. Samples were further fixed by osmium tetroxide and embedded in Epon resin. Thick sections (approximately 1 µm) were cut on an Ultracut E ultramicrotome (Leica Microsystems, Wetzlar, Germany), stained with toluidine blue and examined under a light microscope to ensure the presence of islets. Thin sections (60–80 nm) were then cut from areas of the tissue containing islets, mounted on a Cu/Pd grid (200 mesh), and stained with saturated uranyl acetate and lead citrate. Grids were examined with a Tecnai G2 Spirit electron microscope (FEI Co., Eindhoven, The Netherlands) and representative photographs were taken at either 9300X or 11000X magnification. All chemicals used for electron microscopy were purchased from Canemco, Inc. (Montreal, PQ), unless otherwise stated.

### Oral Glucose Tolerance Test, Glucose-stimulated Insulin Secretion and Insulin Tolerance Test

Mice were fasted for 4 h and then given either an oral glucose gavage (2 g/kg body weight) of a 30% glucose solution or an intraperitoneal injection of 0.7 U/kg body weight of human synthetic insulin (Novolin ge Toronto, Novo Nordisk, Mississauga, Canada). Blood was sampled from the saphenous vein and measured for glucose or insulin before (0 min) and at different timepoints. Blood glucose levels were measured by using a One Touch Ultra glucometer (Life Scan Inc., Burnaby, Canada) and plasma insulin levels were determined by an Insulin Mouse Ultrasensitive enzyme-linked immunosorbent assay (ELISA) (ALPCO Diagnostics, Salem, NH). The area under the curve (AUC) of insulin secretion was calculated after subtraction of basal insulin levels (at 0 min).

### Statistical Analysis

Data are expressed as mean ± SEM. Statistical analysis was performed using Student t-test or two-way ANOVA (GraphPad Prism, GraphPad Software Inc., La Jolla, CA, USA). P values ≤0.05 were considered significant.

## Results

### Neonatal and Adult *Lepr^flox/flox^ RIP-Cre* Islets Exhibit Altered Intracellular Ca^2+^ Signaling in Response to Glucose

**Figure 1:**
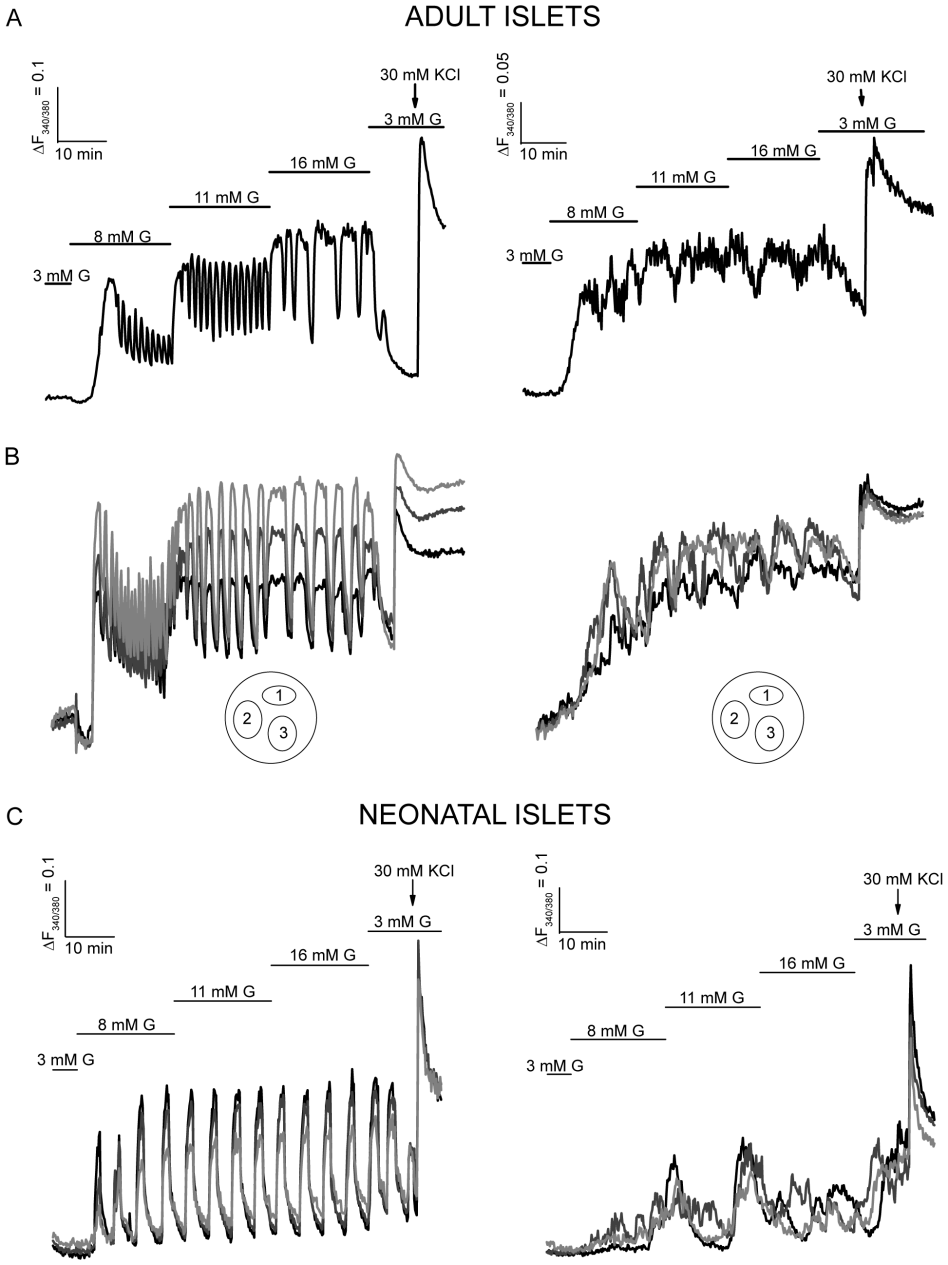
*Lepr^flox/flox^ RIP-Cre* pancreatic β-cells display impaired intracellular Ca^2+^ oscillations in response to glucose. A: [Ca^2+^]_i_ recordings of a *Lepr^flox/flox^* islet (left panel) and *Lepr^flox/flox^ RIP-Cre* islet (right panel) in response to increasing glucose (G) concentrations and potassium chloride (KCl) from adult mice. B and C: Representative [Ca^2+^]_i_ recordings showing three different regions per islet of a *Lepr^flox/flox^* islet (left panel) and a *Lepr^flox/flox^ RIP-Cre* islet (right panel) from adult (B) and neonatal (C) mice. Graphs are representative of 17–20 islets from 3 neonatal mice per group, and 37–38 islets from 3–4 adult mice per group.

*Lepr^flox/flox^ RIP-Cre* mice present difficulties in clearing blood glucose when they undergo an oral glucose tolerance test (OGTT), due at least in part to their impaired ability to secrete insulin in response to an oral glucose bolus [16]. In addition, *in vitro* experiments with perifused *Lepr^flox/flox^ RIP-Cre* islets confirmed the impaired insulin secretion in response to extracellular glucose [16]. GSIS takes place as a result of a sequence of events including glucose uptake and metabolism, membrane depolarization, Ca^2+^ entry through voltage-dependent Ca^2+^-channels and exocytosis of insulin-containing granules [24]. The fact that *Lepr^flox/flox^ RIP-Cre* islets secrete similar insulin amounts in response to KCl compared to their control littermates [16] suggests that the secretory machinery is functional in their β-cells. Since Ca^2+^ is the major intracellular signal regulating insulin secretion, we performed [Ca^2+^]_i_ imaging experiments in intact adult islets (6 weeks of age) in response to increasing glucose concentrations. We observed that while control *Lepr^flox/flox^* islets displayed an organized [Ca^2+^]_i_ oscillatory pattern in response to 8, 11 and 16 mM glucose, *Lepr^flox/flox^ RIP-Cre* islets failed to exhibit normal Ca^2+^ oscillations (Figure 1A). Moreover, thorough analysis of the intracellular Ca^2+^ recordings showed lack of synchrony within 57% of *Lepr^flox/flox^ RIP-Cre* islets whereas all *Lepr^flox/flox^* islets analyzed were well synchronized (Figure 1B).

*Lepr^flox/flox^ RIP-Cre* mice develop obesity and significant differences in body weight can be observed from 3 weeks of age [16]. To rule out the possible contribution of obesity to pancreatic β-cell [Ca^2+^]_i_ signaling we performed [Ca^2+^]_i_ imaging experiments in intact islets isolated from 7–9 day old mice. At that age there are no significant differences in either body weight (5.35±0.617 g for *Lepr^flox/flox^* and 5.54±0.457 g for *Lepr^flox/flox^ RIP-Cre* mice, *P* = 0.807) or in plasma triglyceride levels (1.09±0.26 mM for *Lepr^flox/flox^* and 0.75±0.17 mM for *Lepr^flox/flox^ RIP-Cre* mice, *P* = 0.282). In contrast, these parameters are markedly increased in adult *Lepr^flox/flox^ RIP-Cre* mice compared to Lepr*^flox/flox^* mice [16]. Similar to the observations in the adult islets, we detected an impairment in the oscillatory [Ca^2+^]_i_ pattern in response to glucose accompanied by asynchrony in 87.5% of *Lepr^flox/flox^ RIP-Cre* neonatal islets (Figure 1C). We also observed that, in contrast to adult islets, the intracellular Ca^2+^ oscillations in neonatal β-cells did not vary in width when the extracellular glucose concentration was increased, which could be an indication of immaturity.

### Glucose is Properly Metabolized in *Lepr^flox/flox^ RIP-Cre* Islets

**Figure 2:**
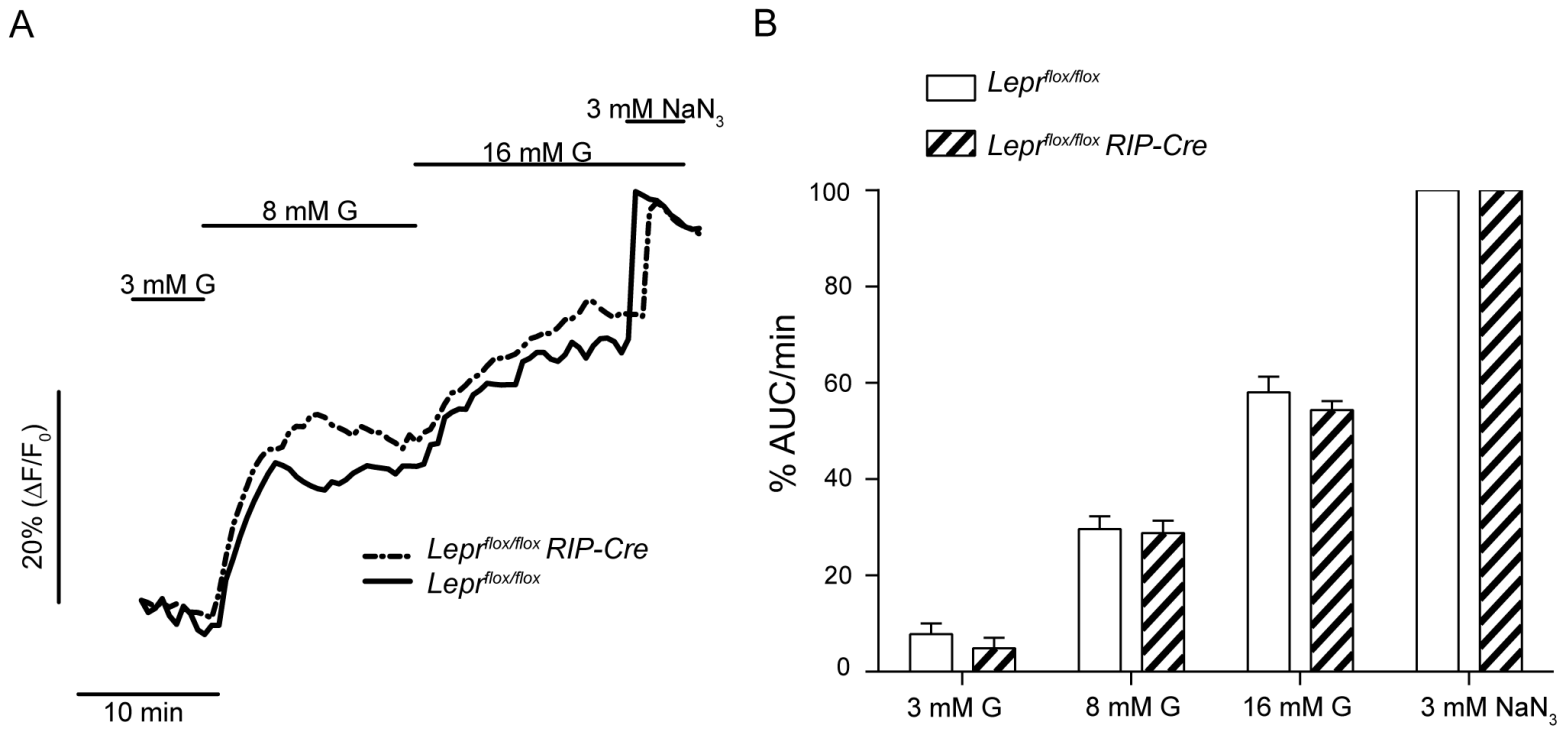
Similar glucose metabolic rates in islets from *Lepr^flox/flox^* and *Lepr^flox/flox^ RIP-Cre* adult mice. A: Two [NAD(P)H]_i_ representative recordings of a *Lepr^flox/flox^* islet (solid line) and a *Lepr^flox/flox^ RIP-Cre* islet (dotted line) in response to glucose (G) and sodium azide (NaN_3_). B: Graph plotting percentage of AUC/min in response to different glucose concentrations and normalized to the maximum reduction level obtained with 3 mM NaN_3_. Data are expressed as mean ± SEM. Statistical analysis was performed using Student t test. Graphs are representative of 32–34 islets from 3 mice per group.

Results obtained from single pancreatic β-cells have demonstrated that prior to Ca^2+^ entry there is an increase of NAD(P)H as a result of glucose being metabolized [25]. To assess whether the lack of leptin signaling affects glucose metabolism in β-cells we measured the autofluorescence of NAD(P)H in 6 week old *Lepr^flox/flox^ RIP-Cre* and control *Lepr^flox/flox^* islets stimulated with increasing extracellular glucose concentrations (Figure 2A). At the end of the experiment islets were exposed to 3 mM sodium azide (NaN_3_) in order to maximize NAD(P)+ reduction. The area under the curve of the increase in fluorescence was calculated for each glucose concentration and presented as the percentage of that obtained with NaN_3_ (Figure 2B). Measurements of NAD(P)H-derived autofluorescence did not reveal differences in the metabolic rates between the two groups, indicating that the impaired insulin response to a glucose stimulus in *Lepr^flox/flox^ RIP-Cre* islets is not due to a failure in glucose metabolism.

### *Lepr^flox/flox^ RIP-Cre* Mice Islets Exhibit Altered Intracellular Ca^2+^ Signaling in Response to Tolbutamide

**Figure 3:**
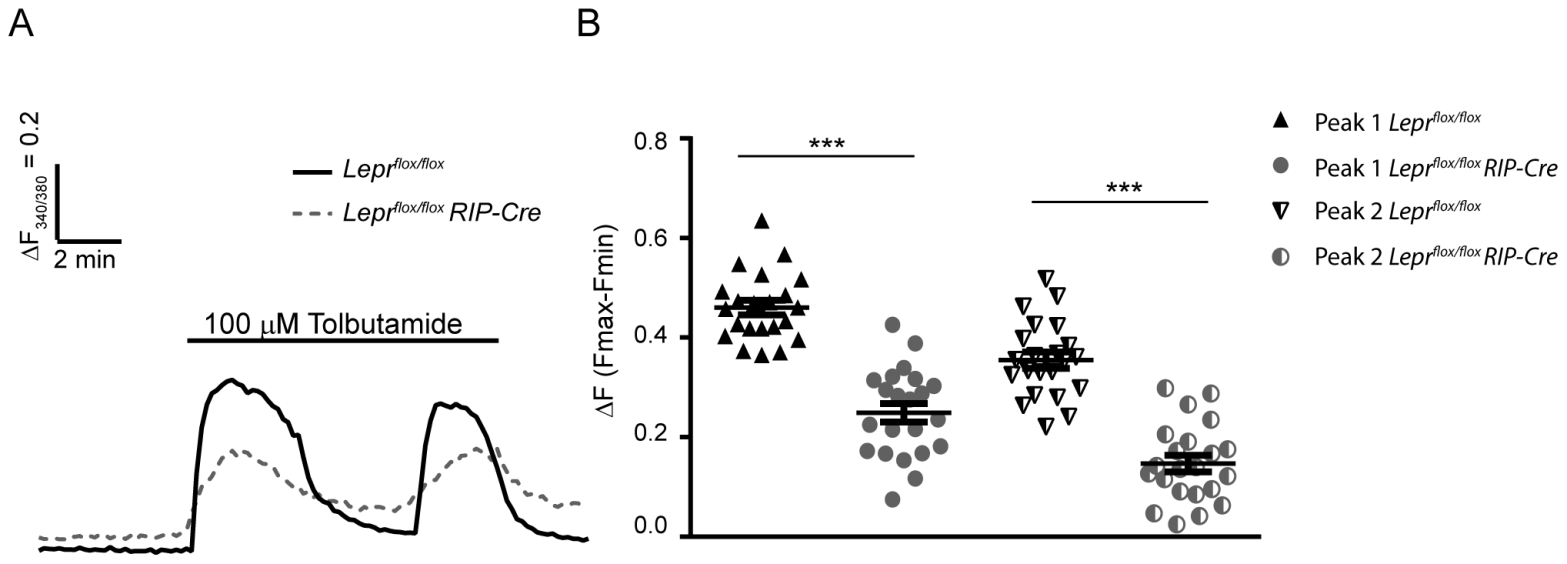
Decreased amplitude in intracellular Ca^2+^ responses to tolbutamide in *Lepr^flox/flox^ RIP-Cre* adult islets. A: Representative recordings from a control *Lepr^flox/flox^* islet (solid line) and a *Lepr^flox/flox^ RIP-Cre* islet (dotted line) in response to tolbutamide. B: Graph plotting ΔFmax-Fmin of each peak in response to tolbutamide in the population of islets that showed two peaks. Data are expressed as mean ± SEM. Statistical analysis was performed using Student t test, *** p<0.0001. Responses are representative of 22 islets from 4 mice per group.

Since glucose is properly metabolized (Figure 2), but intracellular Ca^2+^ oscillations in response to glucose in islets lacking β-cell leptin signaling are impaired (Figure 1A), we next examined the intracellular Ca^2+^ oscillations triggered by another insulin secretatogue. The sulfonylurea tolbutamide blocks β-cell K_ATP_ channels leading to membrane depolarization and subsequent Ca^2+^ entry and insulin secretion [26]. We stimulated 6 week old *Lepr^flox/flox^ RIP-Cre* and *Lepr^flox/flox^* islets in the presence of 100 µM tolbutamide for 10 min and analyzed their [Ca^2+^]_i_ responses. The majority of islets from both transgenic and control mice (62.86 and 64.71%, respectively) displayed two peaks of intracellular Ca^2+^ during the period of stimulation (Figure 3A); peak heights were analyzed and compared (Figure 3B). Our results revealed a significant decrease in both peaks in response to tolbutamide in islets from *Lepr^flox/flox^ RIP-Cre* mice compared to their control *Lepr^flox/flox^* littermates. The remaining islets displayed either 1 peak (8.82% of control islets and 0% of transgenic islets), a sustained increase (26.47% of control islets and 5.71% of transgenic islets) or 3 peaks (0% of control islets and 31.4% of transgenic islets) under tolbutamide stimulation. These results indicate a possible dysfunction of voltage dependent Ca^2+^- or K_ATP_-channels in *Lepr^flox/flox^ RIP-Cre* β-cells.

### Transmission Electron Microscopy Reveals Increased Autophagy in *Lepr^flox/flox^ RIP-Cre* β-cells

**Figure 4:**
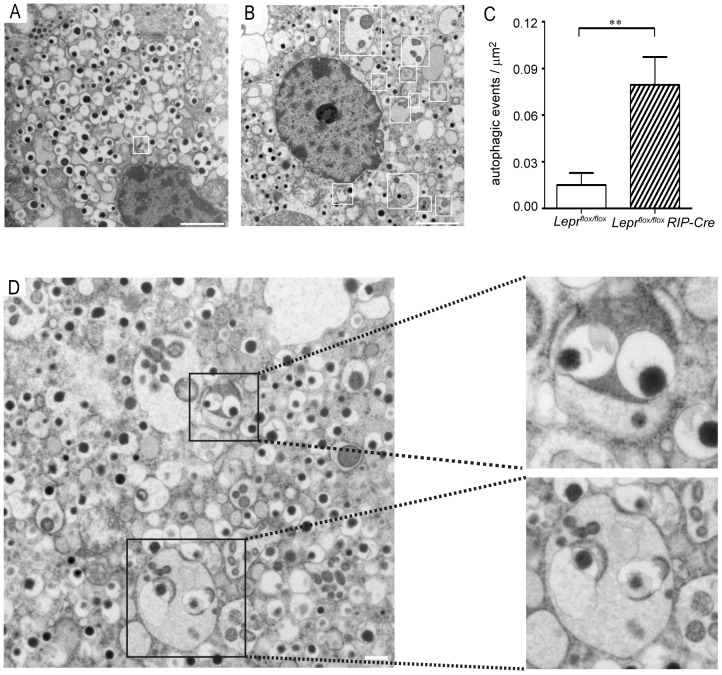
Transmission electron microscopy reveals autophagy within *Lepr^flox/flox^ RIP-Cre* β-cells. Pancreas sections from *Lepr^flox/flox^* (A) and *Lepr^flox/flox^ RIP-Cre* (B and D) mice were analyzed by transmission electron microscopy (magnification 9300X and 11000X) and quantified (C). Multigranular bodies were numerous in *Lepr^flox/flox^ RIP-Cre* β-cells compared to *Lepr^flox/flox^* β-cells (white squares). Events of macroautophagy (Figure 4D, upper inset) and microautophagy (Figure 4D, bottom inset) were captured in *Lepr^flox/flox^ RIP-Cre* β-cells. Scale bar = 2 µm (A and B) and 0.5 µm (D). Micrographs are representative of 3 pancreata analyzed per group. Data are expressed as mean ± SEM. Statistical analysis was performed using Student t test, ** p<0.01.

Intracellular degradation of dense-core insulin secretory granules (β-granules) occurs via autophagy or crinophagy, and these processes are necessary to adequately maintain a balance between the biosynthesis and release of insulin [27], [28]. In the basal state, *Lepr^flox/flox^ RIP-Cre* β-cells hypersecrete insulin [16]. This fact, together with the observed lack of synchrony within *Lepr^flox/flox^ RIP-Cre* islets (Figure 1, B and C), led us to examine the morphology of the islets by transmission electron microscopy. When comparing pancreas micrographs of 6 week old *Lepr^flox/flox^ RIP-Cre* with control *Lepr^flox/flox^* mice we observed a robust increase in granule autophagy within *Lepr^flox/flox^ RIP-Cre* β-cells (0.079±0.017 events/µm^2^, Figure 4B and C), whereas *Lepr^flox/flox^* micrographs showed minimal evidence of these processes (0.015±0.002 events/µm^2^, Figure 4A and C). Autophagy can be mediated by either the formation of an autophagosome that is later fused with a multigranular body (macroautophagy), or by a β-granule being engulfed by a multigranular body in a phagocytotic-like mode (microautophagy) [29]. The autophagic events observed in *Lepr^flox/flox^ RIP-Cre* β-cells are indicative of both autophagy mechanisms. We captured autophagosomes characterized by a double-membrane encapsulating a β-granule (Figure 4D, upper inset), as well as events of microautophagy showing phagocytosis of a β-granule (Figure 4D, bottom inset).

### Normal Glucose Tolerance and Insulin Secretion Despite Disrupted Intracellular Ca^2+^ Signaling in *Lepr^+/+^ RIP-Cre* Mice

**Figure 5:**
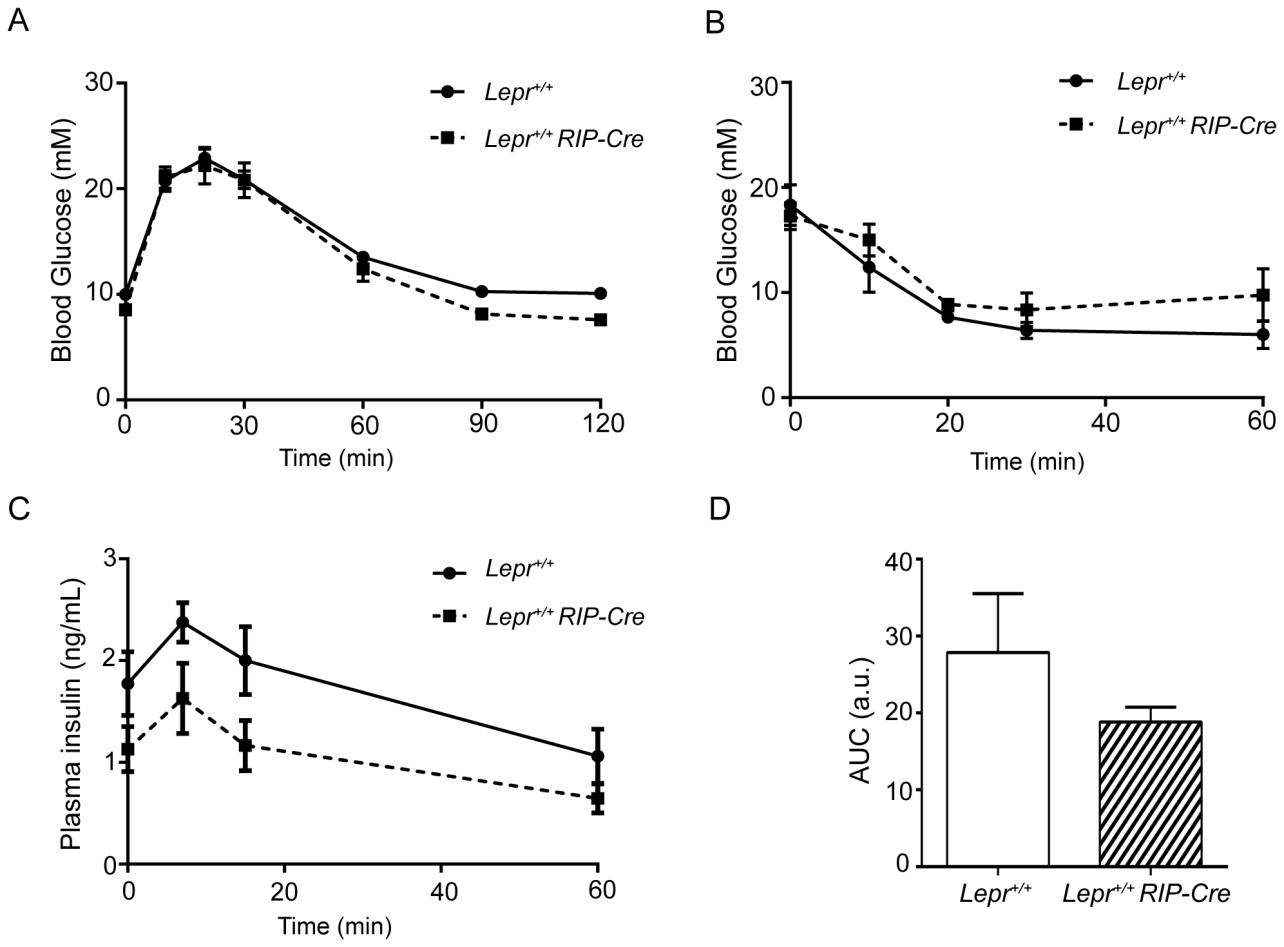
*Lepr^+/+^ RIP-Cre* mice display normal glucose tolerance and insulin secretion. A: OGTT was performed in *Lepr^+/+^* (solid line) and *Lepr^+/+^ RIP-Cre* (dotted line) mice following a dextrose load of 2 g/kg body weight after a 4 hour fast. B: ITT was performed in *Lepr^+/+^* (solid line) and *Lepr^+/+^ RIP-Cre* (dotted line) mice following an intraperitoneal injection of 0.7 U/kg after a 4 hour fast. C: Glucose-stimulated insulin secretion was performed in *Lepr^+/+^* (solid line) and *Lepr^+/+^ RIP-Cre* (dotted line) mice following a dextrose load of 2 g/kg body weight after a 4 hour fast. D: area under the curve (AUC) of the insulin excursion after the oral glucose load calculated after subtraction of basal insulin levels (at time zero).

It has been previously reported that certain transgenic *RIP-Cre* lines have impaired glucose tolerance and insulin secretion [20], although others do not show impairments in glucose homeostasis [16], [21]. We previously characterized the phenotype of the *Lepr^flox/flox^ RIP-Cre* mice and performed glucose tolerance tests in the heterozygous *Lepr^flox/+^ RIP-Cre* group; no differences were observed between this group and the control *Lepr^flox/flox^* group [16]. Since our breeding scheme does not produce *Lepr^+/+^ RIP-Cre* mice, we generated these mice by intercrossing *Lepr^flox/flox^ RIP-Cre* mice with C57BL/6 mice (homozygous for the *Lepr+* allele), and studied their glucose metabolism. There were no differences between *Lepr^+/+^ RIP-Cre* and *Lepr^+/+^* mice in response to an OGTT or during an insulin tolerance test (Figure 5A and B). Basal plasma insulin levels were also not found to be significantly different (*P* = 0.131) and glucose-stimulated insulin secretion was similar between *Lepr^+/+^ RIP-Cre* and *Lepr^+/+^* mice (Figure 5C and D), although a slight decrease in insulin levels at fasting state and in response to glucose was observed in the transgenic group.

**Figure 6:**
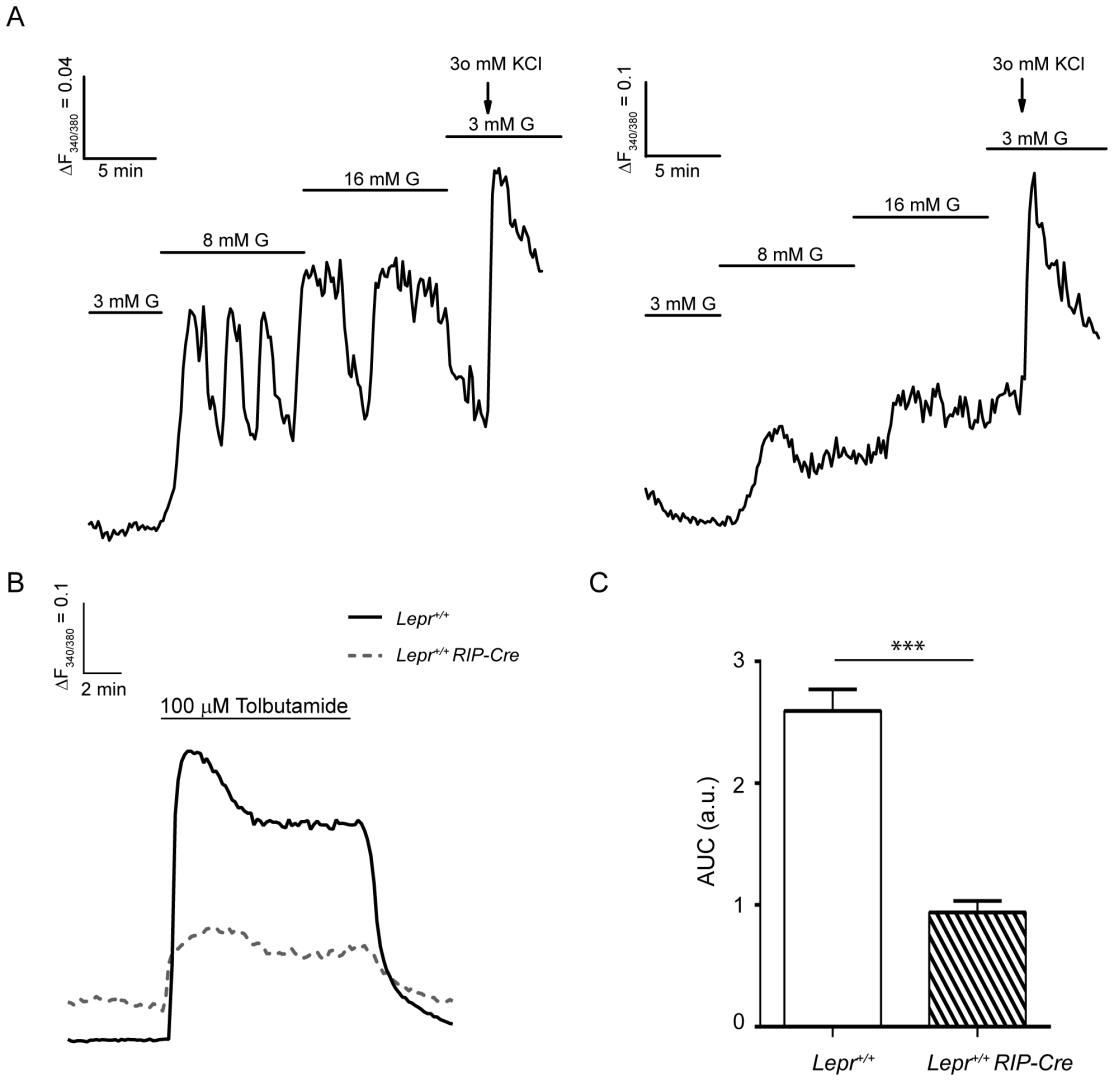
*Lepr^+/+^ RIP-Cre* mice present impaired β-cell Ca^2+^ signaling in response to glucose and tolbutamide. A: Representative [Ca^2+^]_i_ recordings of a *Lepr^+/+^* islet (left panel) and *Lepr^+/+^ RIP-Cre* islet (right panel) in response to increasing glucose (G) concentrations and potassium chloride (KCl) from 5–6 week old mice. B: Representative [Ca^2+^]_i_ recordings of a *Lepr^+/+^* islet (solid line) and *Lepr^+/+^ RIP-Cre* islet (dotted line) in response to tolbutamide. C: Graph plotting the AUC of the Ca^2+^ transients during the stimulation with tolbutamide. Data are expressed as mean ± SEM. Statistical analysis was performed using Student t test. *** p<0.0001. Graphs are representative of 18–20 islets from 3 mice per group (in response to glucose) and 16–27 islets from 2–3 mice per group (in response to tolbutamide).

To more carefully assess the contribution of the Cre transgene to the β-cell phenotype of the *Lepr^flox/flox^ RIP-Cre* mice, we performed Ca^2+^ imaging in islets isolated from *Lepr^+/+^ RIP-Cre* mice. Unexpectedly, we did observe an abnormal intracellular Ca^2+^ signaling pattern in islets from 5–6 week old *Lepr^+/+^ RIP-Cre* mice in response to glucose (Figure 6A), which resembled that observed in the *Lepr^flox/flox^ RIP-Cre* islets (Figure 1A). Intercellular asynchrony within the islets was found in 30% of the *Lepr^+/+^ RIP-Cre* islets while all the *Lepr^+/+^* islets displayed synchrony. We also imaged the intracellular Ca^2+^ in response to tolbutamide. The majority of islets from both transgenic and control mice (79.4 and 69.6%, respectively) displayed a sustained transient of intracellular Ca^2+^ during the period of stimulation, with a significant decrease in the AUC of *Lepr^+/+^ RIP-Cre* group (Figure 6B and C). These results indicate that Cre expression itself affects β-cell function *in vitro*, independently of the presence of a floxed gene, on this genetic background. However, this abnormality does not appear to affect whole body glucose homeostasis, although it could be responsible for the trend towards lower insulin levels in response to glucose observed during the glucose-stimulated insulin secretion (Figure 5C).

## Discussion

This study describes morphological and functional abnormalities found in insulin-secreting β-cells from mice specifically lacking leptin receptors by Cre-loxP recombination. For the purpose of knocking down genes in pancreatic β-cells, *RIP-Cre* mice have been widely crossed with mice carrying floxed genes [16], [20], [30], [31]. However, it was noted by Lee *et al*. that many mice generated with the Cre-loxP technique by using *RIP-Cre* mice were glucose intolerant, which prompted the authors to perform parallel OGTTs in *RIP-Cre* mice originating from three independent laboratories and having different genetic backgrounds. They observed impaired glucose tolerance in all three lines [20]. In contrast, *RIP-Cre* mice were found to be glucose tolerant in other studies [21], [30], [32], which suggests that the genetic background of each strain is important for the *RIP-Cre* phenotype, at least in terms of glucose homeostasis. In the past we employed a *RIP-Cre* line to generate *Lepr^flox/flox^ RIP-Cre* mice [16]. The OGTT performed in heterozygous *Lepr^flox/+^ RIP-Cre* mice showed a normal glucose excursion ruling out a possible effect of Cre expression on glucose clearance in our mice [16]. Furthermore, the OGTT and GSIS carried out in *Lepr^+/+^ RIP-Cre* mice in the present study confirm the normal glucose metabolism of these mice. Hence, we employed our *Lepr^flox/flox^ RIP-Cre* model to better understand the cellular pathways and mechanisms that link diabetes and obesity.

Intra-islet cell communication, largely achieved through gap-junctions, seems to be required for proper insulin secretion [33]. Glucose-stimulated Ca^2+^ oscillations are synchronized within the islets and are accompanied by synchronized oscillations of insulin release [34], [35]. In our experiments we found that *Lepr^flox/flox^ RIP-Cre* islets failed to exhibit normal intracellular Ca^2+^ oscillations in response to glucose. In addition, asynchronous oscillations were detected within different regions in 57% of *Lepr^flox/flox^ RIP-Cre* islets while control *Lepr^flox/flox^* islets were well synchronized and displayed an organized [Ca^2+^]_i_ oscillatory pattern. Reportedly, islets from animal models that lack leptin signaling such as *db/db* and *ob/ob* mice display intracellular Ca^2+^ abnormalities in their β-cells, including lack of synchrony and impairments in their response to glucose [36], [37]. In order to determine whether or not Cre expression could influence the effects observed on Ca^2+^ signaling we imaged intracellular Ca^2+^ in *Lepr^+/+^ RIP-Cre* islets in response to glucose. Surprisingly, we observed abnormal Ca^2+^ oscillations in response to glucose, and 30% of the *Lepr^+/+^ RIP-Cre* islets displayed asynchrony. This suggests that Cre expression alone may partially contribute to the poor intracellular communication observed in 57% of *Lepr^flox/flox^ RIP-Cre* islets.

For these studies we also performed intracellular Ca^2+^ imaging in isolated neonatal islets; perhaps the first β-cell Ca^2+^ recordings reported at this young age. We noted defects similar to the adult *Lepr^flox/flox^ RIP-Cre* islets when intracellular Ca^2+^ was imaged in intact islets isolated from neonatal *Lepr^flox/flox^ RIP-Cre* mice. Interestingly, a higher percentage of neonatal *Lepr^flox/flox^ RIP-Cre* islets (85%) displayed asynchrony compared to adult islets (57%). Knockout models tend to activate compensatory mechanisms; hence the reduced asynchrony in *Lepr^flox/flox^ RIP-Cre* islets with age could be the result of an adaptive response to the lack of leptin signaling. Another consideration might be that the possible immature state of the neonatal islets exacerbates the lack of synchrony. Notably, in control neonatal islets, we observed regular Ca^2+^ oscillations in response to extracellular glucose that, unlike adult islets, did not increase in width as glucose concentration increased (from 8 to 16 mM). We therefore interpreted their intracellular Ca^2+^ pattern as “immature”. Recently, Blum *et al*. showed that islets from mice older than P9 respond as “mature” β-cells and that β-cell maturation occurs between P2 and P9 [38]. Our results with islets from neonate mice P7 to P9 suggest that the maturity process in terms of Ca^2+^ signaling has not been fully achieved by P9.

Whether the abnormal intracellular Ca^2+^ pattern in the *Lepr^flox/flox^ RIP-Cre* β-cells is a result of impaired glucose metabolism was also analyzed. We measured the autofluorescence emitted by NAD(P)H in response to glucose and did not find differences between the control and the experimental group, suggesting that the defects in Ca^2+^ signaling are likely due to a dysfunction of ion channels (voltage dependent Ca^2+^- or K_ATP_-channels). Parallel Ca^2+^ imaging experiments in response to tolbutamide strongly support this possibility. The sulphonylurea tolbutamide closes K_ATP_ channels, which results in membrane depolarization and Ca^2+^ entry. When *Lepr^flox/flox^ RIP-Cre* islets were stimulated with tolbutamide a significant reduction in the Ca^2+^ influx was found compared to control *Lepr^flox/flox^* islets. Similar observations have been reported from leptin receptor deficient *db/db* β-cells, which show a diminished response to tolbutamide [39]. In addition, defective electrical activity characterized by altered [Ca^2+^]_i_-activated K^+^ permeability or decreased L-type Ca^2+^ currents has been described in islets from different diabetic models [40], [41]. However, we examined whether or not the defects in the response to tolbutamide are influenced by the expression of the Cre transgene and the results confirmed that Cre expression itself alters intracellular Ca^2+^ signaling in β-cells.

Transmission electron micrographs of *Lepr^flox/flox^ RIP-Cre* pancreata revealed a drastic increase in autophagy in the insulin-secreting cells. Upregulated autophagy has been observed in β-cells from human patients with type 2 diabetes [42], as well as in β-cells from mice fed with a high fat diet [43]. We previously demonstrated that *Lepr^flox/flox^ RIP-Cre* mice are hyperinsulinemic, glucose intolerant and insulin resistant [16]. Therefore, we speculate that the dramatic upregulation of autophagy pathways in β-cells from *Lepr^flox/flox^ RIP-Cre* mice may represent an inherent imbalance in the production and secretion of insulin granules. The autophagic degradation of insulin granules observed in this study may be an adaptive mechanism aimed to maintain appropriate intracellular insulin stores. Notably, our studies revealed that unlike *Lepr^flox/flox^ RIP-Cre* mice, the *Lepr^+/+^ RIP-Cre* group displayed normal glucose and insulin tolerance, as well as normal plasma insulin levels. Hence, we do not believe that the dramatic upregulation of autophagy observed in β-cells lacking functional leptin receptors resulted from Cre expression.

Together, these results indicate that β-cells lacking leptin receptors by Cre-loxP recombination display functional and morphological alterations. Since we have also observed intracellular Ca^2+^ defects in *Lepr^+/+^ RIP-Cre* control islets it is difficult to discern the extent of the effect that may be produced by expression of Cre recombinase as opposed to the lack of leptin signaling. As discussed above, we do believe that the lack of functional leptin receptors does disrupt the synchrony within islets and also promotes intracellular β-granule degradation. However, given the clear effects of the Cre transgene alone, another model system will need to be used to confirm these findings. Interestingly, despite having defective Ca^2+^ signaling in their β-cells, the *RIP-Cre* mice employed in this study showed normal glucose homeostasis. Thus, caution should be taken when using transgenic mice even in those cases where the Cre transgenic model shows no effect *in vivo*. Therefore, our work highlights the need to generate improved Cre-driver models or new genetic tools to induce specific Cre recombination in β-cells without affecting the function of this cell population.
